# A versatile system for fast screening and isolation of *Trichoderma reesei* cellulase hyperproducers based on DsRed and fluorescence-assisted cell sorting

**DOI:** 10.1186/s13068-018-1264-z

**Published:** 2018-09-24

**Authors:** Fei Gao, Zhenzhen Hao, Xianhua Sun, Lina Qin, Tong Zhao, Weiquan Liu, Huiying Luo, Bin Yao, Xiaoyun Su

**Affiliations:** 1 Key Laboratory for Feed Biotechnology of the Ministry of Agriculture, Feed Research Institute, Chinese Academy of Agricultural Sciences, No. 12 South Zhongguancun Street, Beijing, 100081 People’s Republic of China; 2 College of Biological Sciences, China Agricultural University, Beijing, 100193 China; 3 Institute of Microbiology, Chinese Academy of Sciences, Beijing, 100101 China

**Keywords:** *Trichoderma reesei*, DsRed, FACS, Cellulase, High-throughput screening

## Abstract

**Background:**

In the biofuel industry, cellulase plays an indispensable role in hydrolyzing cellulose into fermentable glucose. *Trichoderma reesei* is a popular filamentous fungus with prominent ability to produce cellulase. While classical mutagenesis and modern multiplex genome engineering are both effective ways to improve cellulase production, successful obtaining of strains with improved cellulase-producing ability requires screening a large number of strains, which is time-consuming and labor intensive.

**Results:**

Herein, we developed a versatile method coupling expression of the red fluorescence protein (DsRed) in *T. reesei* and fluorescence-assisted cell sorting (FACS) of germinated spores. This method was first established by expressing DsRed intracellularly under the control of the major cellulase *cbh1* promoter in *T. reesei*, which allowed us to rapidly isolate cellulase hyperproducers from *T. reesei* progenies transformed with a dedicated transcriptional activator *ace3* and from an atmospheric and room temperature plasma-created mutant *T. reesei* library. Since intracellularly expressed DsRed was expected to isolate mutations mainly affecting cellulase transcription, this method was further improved by displaying DsRed on the *T. reesei* cell surface, enabling isolation of strains with beneficial genetic alterations (overexpressing *hac1* and *bip1*) affecting regulatory stages beyond transcription. Using this method, *T. reesei* cellulase hyperproducers were also successfully isolated from an *Agrobacterium*-mediated random insertional mutant library.

**Conclusions:**

The coupled DsRed-FACS high-throughput screening method proved to be an effective strategy for fast isolation of *T. reesei* cellulase hyperproducers and could also be applied in other industrially important filamentous fungi.

**Electronic supplementary material:**

The online version of this article (10.1186/s13068-018-1264-z) contains supplementary material, which is available to authorized users.

## Background

*Trichoderma reesei* is a saprophytic wood-decaying filamentous fungus with prominent ability to produce cellulase. The maximal extracellular protein concentration of *T. reesei* cellulase has been reported to reach 100 g/L [1]. This ascomycete is, therefore, also regarded as a promising host for producing heterologous proteins. Indeed, a vast number of valuable enzymes and pharmaceutical proteins have been produced in this organism, which includes the *Phlebia radiate* laccase [2], *Hormoconis resinae* glucoamylase [3], barley endopeptidase B [4], *Aspergillus niger* acid phosphatase [5], lipase [6], glucose oxidase [7], endo-mannanase [8], antibodies, and interferons [9, 10].

On the route towards lignocellulosic biofuels, cellulase plays an indispensable role by hydrolyzing cellulose into fermentable glucose. *T. reesei* has long been used to produce cellulase and currently is still one popular microbe [11]. Varying strategies have been utilized to improve cellulase production in *T. reesei*, mainly by engineering the *T. reesei* strains and modifying the fermentation processes. Cellulase (and other kinds of enzymes or proteins) is synthesized from amino acid precursors. Therefore, the concept of metabolic engineering can be employed to improve cellulase expression and secretion in *T. reesei* basically by means of random mutagenesis and genetic engineering [12]. To improve production of cellulase widely useful in the biofuel industries, chemical and physical mutagenesis were initially carried out, which successfully generated hyperproducer mutants such as RUT-C30 [13] and CL-847 [14]. Additionally, *Agrobacterium*-mediated random insertional disruption of chromosomal genes was also successful in generating *T. reesei* hyperproducers [15]. Genetic engineering by overexpressing selected genes stimulating cellulase expression [7, 16] and removing the ones repressing cellulase expression [17, 18] controlling transcription, translation, and secretion as well as intracellular redox balance and cell metabolism [19–22] have become two common strategies to improve cellulase production in *T. reesei*. It is noticed that multiplex genome engineering, i.e., manipulating more than one gene at a time, creates rich biological diversity from which a cellulase hyperproducer can be isolated [16]. By including more genes to manipulate, this biodiversity may even ascend, increasing the possibility to obtain hyperproducers. However, the concurring higher complexity demands a larger amount of transformants to be analyzed.

Both random mutagenesis and genetic engineering methods require considerable time and labor in screening. For *T. reesei*, this challenge is further complicated by the facts that *T. reesei* is multicellular and filamentous, cellulase is extracellularly expressed, and integration site and copy numbers largely affect gene expression level [23–25]. These characters dwarfed the endeavors to establish high-throughput screening methods. Coupling expression of green fluorescence protein (GFP) and fluorescence-assisted cell sorting (FACS) achieved isolation of *T. reesei* cellulase hyperproducers rapidly [26]. However, the expression of GFP is not easily observed without a fluorescence microscope. More importantly, this method cannot identify genetic alterations favoring improved secretion, which is elegantly regulated, but critical for cellulase expression [22, 27]. On the other hand, time can also be saved by scaling down from traditional shake flask to microtiter plate culture; however, the quantity of strains to be screened is still large [28]. Herein, we sought to overcome these bottlenecks and establish a new high-throughput screening method Specifically, we used the red fluorescence protein DsRed as a reporter molecule and demonstrated that the intracellularly expressed, and more importantly, surface-displayed DsRed coupled with FACS can be used for high-throughput screening of cellulase hyperproducers from *T. reesei* progeny libraries generated by random mutagenesis or genetic engineering. It is expected that our results may provide a robust engineering framework for future efforts to engineer *T. reesei*, and other industrially important filamentous fungi as well, for enhanced secretion of cellulase and other valuable proteins.

## Results

### Constructing the plasmids for intracellular expression and surface-display of DsRed

**Fig. 1:**
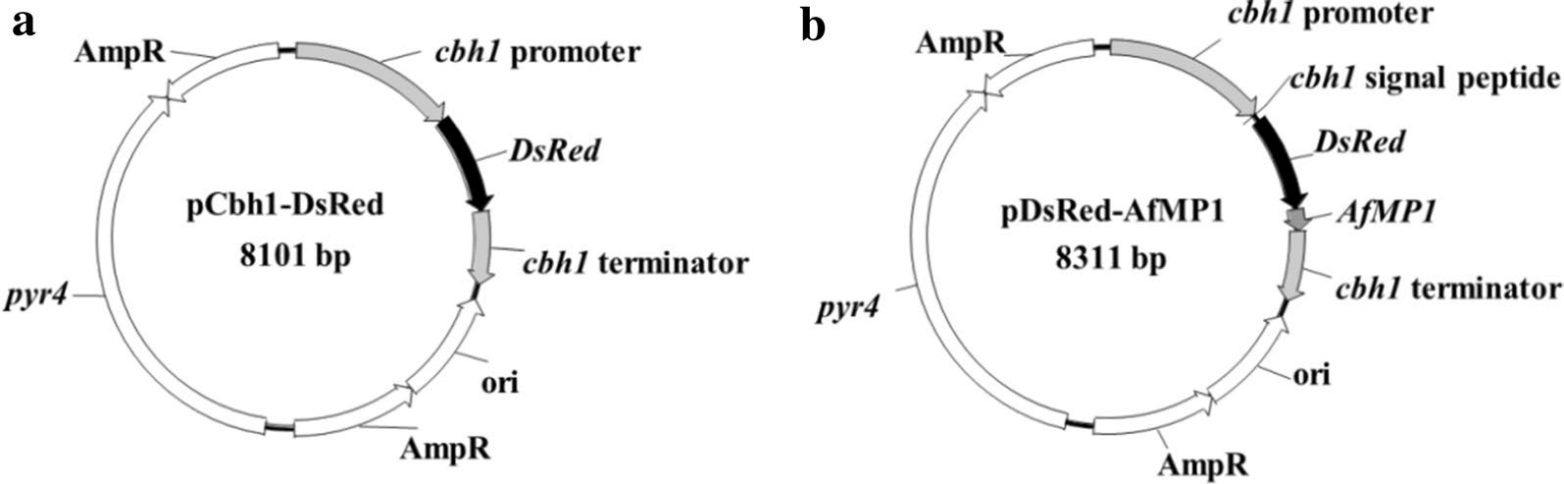
Schematic diagrams of the plasmids for expressing *DsRed*. **a** The pCbh1-DsRed plasmid for expressing DsRed intracellularly. **b** The pDsRed-AfMP1 plasmid for displaying DsRed on *T. reesei* cell surface. For both plasmids, the strong inductive *cbh1* promoter was used. *Af*MP1: the C-terminal GPI anchor from the *A. fumigatus* MP1 protein

DsRed is a coral red fluorescence protein with an excitation wavelength of 558 nm and an emission wavelength of 583 nm, respectively [29]. To correlate the expression of *DsRed* to that of the *T. reesei* cellulase, the *DsRed* gene should be placed under the control of a major cellulase promoter. Cellobiohydrolase I (CBH1) is the highest expressed cellulase in *T. reesei*, accounting up to 50–60% of secreted proteins. Therefore, the codon-optimized *DsRed* gene was ligated downstream of the strong inducible *cbh1* promoter for intracellular expression (Fig. 1a). Surface-display of proteins has been frequently studied in bacteria [30] and yeasts [31], but gains much less attention in filamentous fungi. The *Aspergillus fumigatus* MP1 (*Af*MP1) is a cell wall-attached galactomanno-protein with a clearly defined C-terminal glycosylphosphatidylinositol (GPI) anchor signal sequence [32]. For surface-display of DsRed, the genes encoding the CBH1 signal peptide, DsRed, and the *Af*MP1 GPI anchor signal (GGSGSGSGSSTGTATASTSTNLLSTGAASKEHFSYSLGGAVVAAAIAVA) were in-frame fused. The chimeric gene was also placed between the *cbh1* promoter and terminator (Fig. 1b).

### Intracellular expression of DsRed dictates isolation of genetically engineered *T. reesei* cellulase hyperproducers

**Fig. 2:**
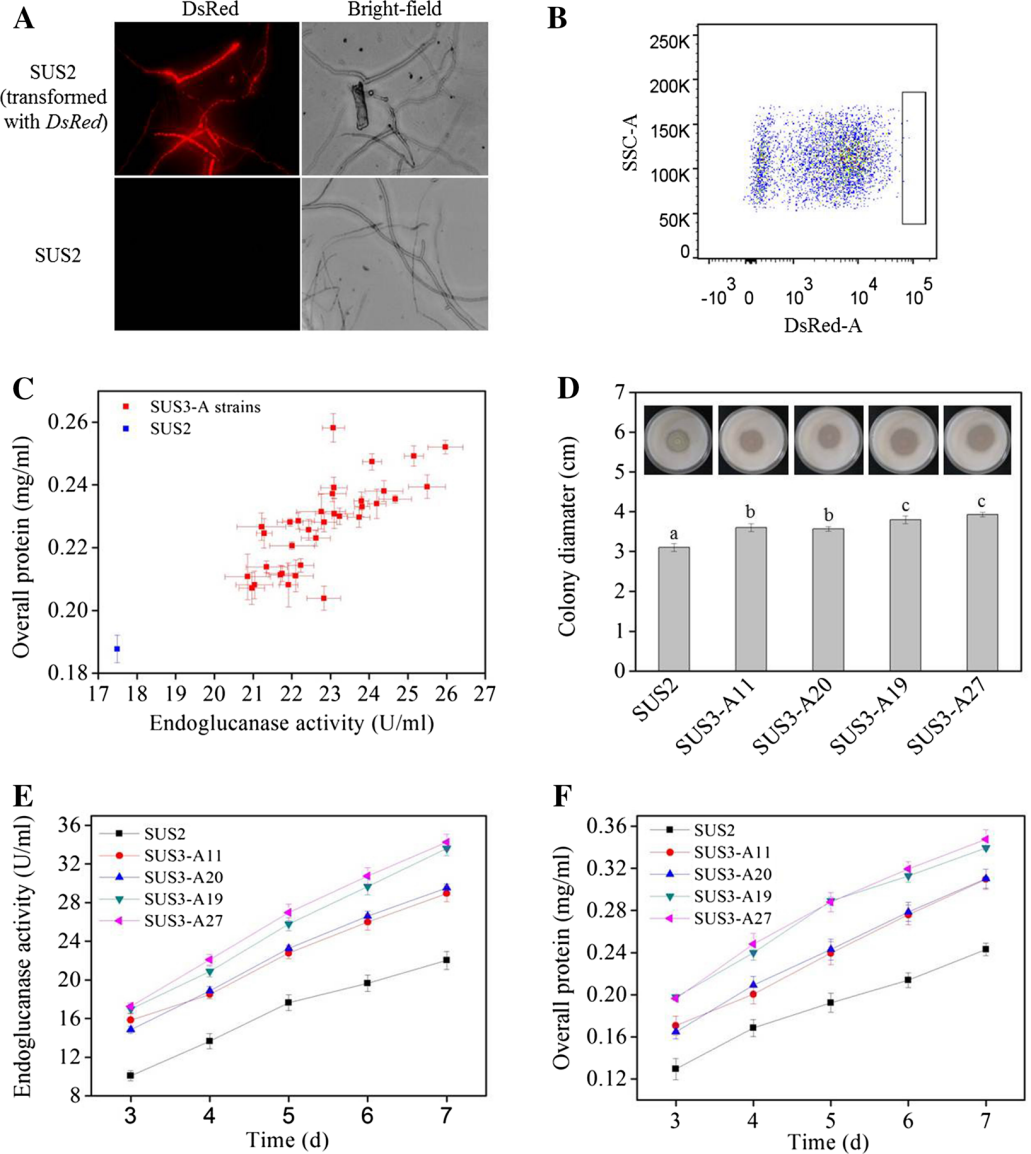
Isolation of cellulase hyperproducers from *ace3*-transformants by FACS using the intracellularly expressing DsRed as an indicator. **A** Microscopic observation of the hyphae of *T. reesei* expressing intracellular DsRed and its parental strain SUS2. Mycelia were harvested from *T. reesei* cultivated at 28 °C for 24 h in MM-lactose/sophorose. **B** Flow cytometry sorting of germlings with the brightest DsRed signal (top 0.1%) from *ace3*-transformants. The area in box was the gating strategy used to delineate the brightest DsRed signal cell populations. **C** Preliminary assessment of endoglucanase producing and protein secretion ability of *ace3*-transformants in shake flask fermentation. **D** The cellulose-hydrolyzing halos formed by isolated representative *ace3*-transformants. Different letters (^a,^ ^b,^ and ^c^) mean that there are significant difference between the colony diameters (*p *< 0.05). **E**, **F** Representative *ace3*-transformants isolated in FACS produced more endoglucanase (**E**) and secreted more proteins (**F**)

The plasmid encoding the codon-optimized *DsRed* under the control of *cbh1* promoter was transformed into *T. reesei* SUS2. *DsRed* was successfully expressed in *T. reesei* and red fluorescence appeared to be evenly distributed in the hyphae cytosome, which could be clearly visualized under fluorescence microscope (Fig. 2A). High level expression of *DsRed* resulted into change of the colony color from pale white to red on a MM-lactose plate, which could even be observed by naked eyes (Additional file 1). Next, we tested whether higher level of *DsRed* expression incurred by genetic modification of *T. reesei* would be positively correlated with higher cellulase production. For this purpose, the transcriptional activator *ace3* was constructed downstream of the strong constitutive *pdc1* promoter to obtain pPdc1-ace3 (Additional file 2). This plasmid was transformed into a uridine-auxotrophic derivative of the *DsRed*-expressing SUS2 transformant, namely SUS3. Note that SUS2 and SUS3 have nearly identical cellulase-producing abilities (data not shown). Overexpression of *ace3* is known to stimulate cellulase expression in *T. reesei* by two- to fourfold [21]. Thus, 40 spores with highest red fluorescence signal (top 0.1%) were collected for further analyses (Fig. 2B). Since SUS3 (and its parent strain SUS2) is a dedicated strain for *egl2*- overexpression, we measured the endoglucanase activity and used it as a cellulase indicator. All these selected *ace3*-transformants behaved better in preliminary flask fermentation, producing more endoglucanase as well as extracellular proteins (Fig. 2C).

SUS2 and four representative *ace3*-transformants with moderately to highly enhanced cellulase-producing ability were grown on cellulose plates for comparison of halos (an indicator of cellulose hydrolysis rate, roughly representing the cellulase activity). All *ace3*-transformants formed a halo (diameter: 3.50–3.95 cm) larger than that of SUS2 (diameter: 3.20 cm), indicative of improved cellulase producing ability (Fig. 2D). In flask fermentation, these four strains exhibited higher endoglucanase activities and protein concentrations from day 3 post-Avicel induction (Fig. 2E, F). These results indicated that the intracellular expressed DsRed under the control of *cbh1* promoter could be used to dictate selection of the *T. reesei* high cellulase producers. Using quantitative PCR, the gene copy numbers of *ace3* in the four transformants were determined to be two for SUS3-A11, SUS3-A19, and SUS3-A20 and three for SUS3-A27, higher than one in the parental strain (data not shown).

### Rapid isolation of *T. reesei* cellulase hyperproducers generated from ARTP random mutagenesis

Through successful isolation of *ace3*-transformants with improved cellulase producing ability, the validity of the high-throughput screening method was demonstrated. However, we realized that random mutagenesis has been used as an effective way for *T. reesei* strain improvement [14] and is still being used widely. Indeed, most industrial strains are mutagenized derivatives of the QM6a strain [13]. Therefore, we tested if this DsRed-based FACS method could also be used for isolation of cellulase hyperproducers from a randomly mutagenized *T. reesei* library.

**Fig. 3:**
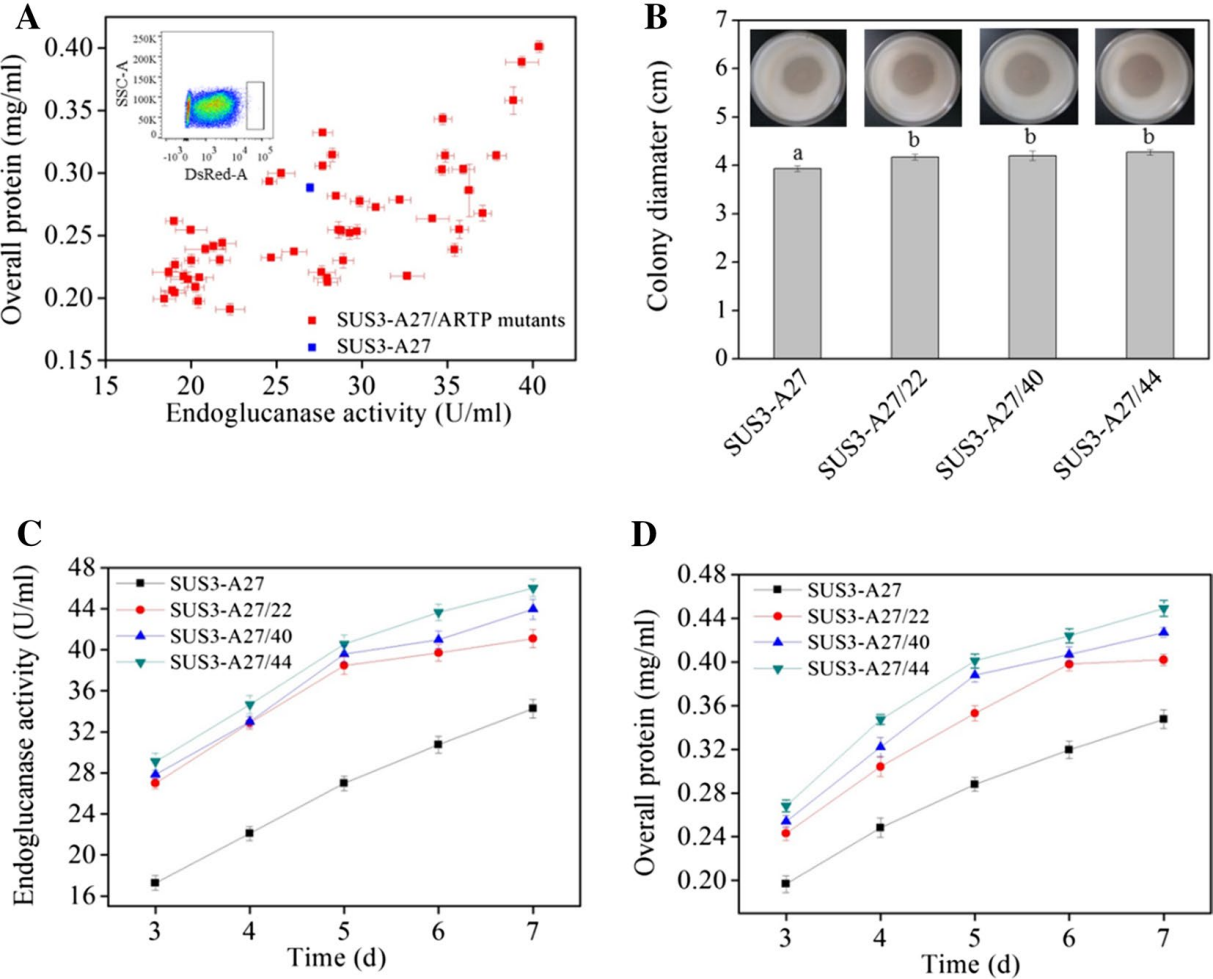
Isolation of cellulase hyperproducers from an ARTP-generated *T. reesei* mutant library using FACS directed by intracellularly expressed DsRed. **A** Preliminary assessment of endoglucanase producing and protein secretion ability of isolated *T. reesei* strains. The *T. reesei* spores were mutagenized using ARTP. Germinated spores with the brightest DsRed signal (top 0.1%) were isolated by FACS (internal illustration). The area in box was the gating strategy used to delineate the brightest DsRed signal cell populations. **B** Representative ARTP-mutagenized strains isolated by FACS displayed larger cellulose hydrolysis halos than the parent strain. Different letters (^a^ and ^b^) mean that there are significant difference between the colony diameters (*p *< 0.05). **C**, **D** Representative ARTP-mutagenized strains isolated by FACS produced more endoglucanase (**C**) and secreted more proteins (**D**)

The atmospheric and room temperature plasma mutation system, or ARTP, uses radio-frequency atmospheric-pressure glow discharge plasma jets to create mutations in the DNA sequence and has been used to mutate more than 40 kinds of microorganisms including bacteria, fungi, and microalgae [33]. SUS3-A27, the best strain of the *ace3*-transformants, was used for mutagenesis. The ARTP-treated spores were grown for 14 h in MM-lactose/sophorose liquid medium for germination. This culture period allows expression of cellulase genes (and here DsRed also) [34] but precludes formation of long intertwining hyphae clog, which would be troublesome for flow cytometry analysis. Fifty-one germinated spores with highest red fluorescence signals were picked out from FACS (Fig. 3A). Unlike the *ace3*-transformation, some of the sorted mutants behaved poorer than the parent strain in the preliminary screening in flask cultivation (Fig. 3A). This indicated that, stronger red fluorescence signal at the early stage of germination for these mutants from random mutagenesis do not necessarily parallel with higher cellulase producing ability. In spite of this inconsistence, certain mutants exhibited higher cellulase activity as well as overall protein concentrations than those of the parent strain (Fig. 3A). Three representative mutant strains (SUS3-A27/22, SUS3-A27/40, and SUS3-A27/44) were used for further detailed analyses. These mutants formed cellulose-hydrolyzing halos with a diameter of 4.15–4.30 cm, respectively, larger than that of SUS3-A27 (3.95 cm) on MM-Avicel plate (Fig. 3B). In addition, on the 7th day post-induction, SUS3-A27/22, SUS3-A27/40, and SUS3-A27/44 produced 41.1, 43.9, and 46.0 U/ml endoglucanase and 0.41, 0.43, and 0.45 mg/ml extracellular proteins, respectively, which were all higher than the values of SUS3-A27 (34.2 U/ml for endoglucanase activity, Fig. 3C) and 0.35 mg/ml (for extracellular protein concentration, Fig. 3D).

### Surface-display of DsRed enabled identifying genetic alterations beneficial for cellulase secretion

The intracellularly expressed DsRed hardly allows identification of beneficial mutations or genetic modifications affecting the stages beyond transcription. However, this obstacle could be overcome if the expressed DsRed reporter protein also undergoes the secretory pathway while being attached to the cell. This can be achieved by displaying DsRed on the *T. reesei* cell surface. For this purpose, we fused the *DsRed* gene in frame between the sequences coding for the CBH1 signal peptide and *Af*MP1 GPI anchor. The CBH1 signal peptide will lead the DsRed protein through the secretory pathway and the covalently linked GPI anchor can keep DsRed attached to the cell surface. These traits are expected to facilitate high-throughput isolation of cellulase hyperproducers generated by genetic alterations at any regulatory stages.

**Fig. 4:**
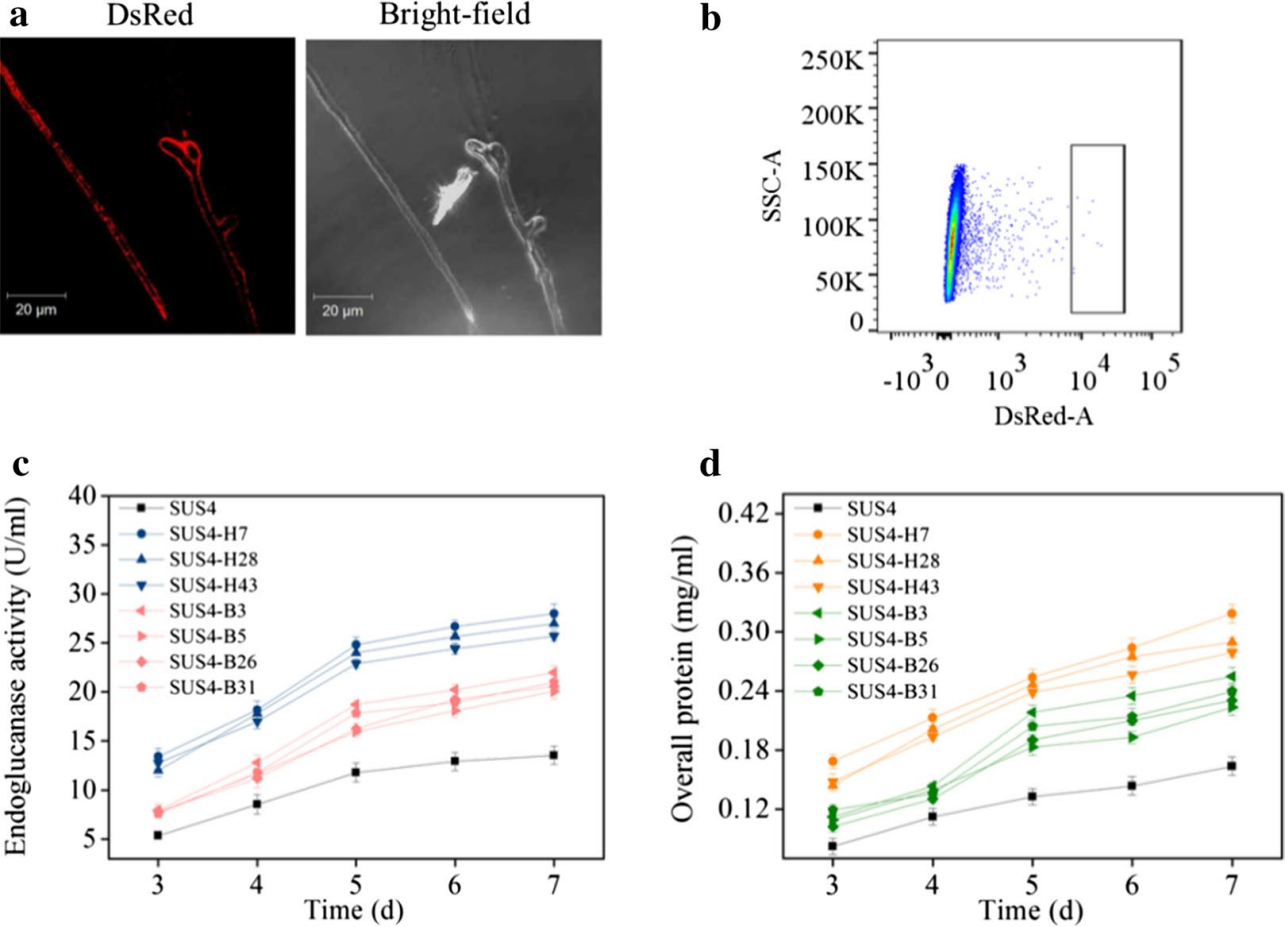
Isolation of cellulase hyperproducers from *T. reesei* transformed with a mixture of six plasmids by FACS directed by surface-displayed DsRed. **a** Microscopic observation of the *T. reesei* hyphae displaying DsRed on cell surface. *T. reesei* was cultivated at 28 °C for 24 h in MM-lactose/sophorose. **b** FACS isolation of germinated spores from *T. reesei* surface-displaying DsRed. *T. reesei* was transformed with a pool of six plasmids for overexpressing *bip1*, *hac1*, *ftt1*, *sso2*, *sar1*, and *ypt1*, respectively. Sixty germlings with the brightest DsRed signal (top 0.1%) were isolated. The area in box was the gating strategy used to delineate the brightest DsRed signal cell populations. **c**, **d** Representative strains isolated by FACS produced more endoglucanase (**c**) and secreted more proteins (**d**)

**Fig. 5:**
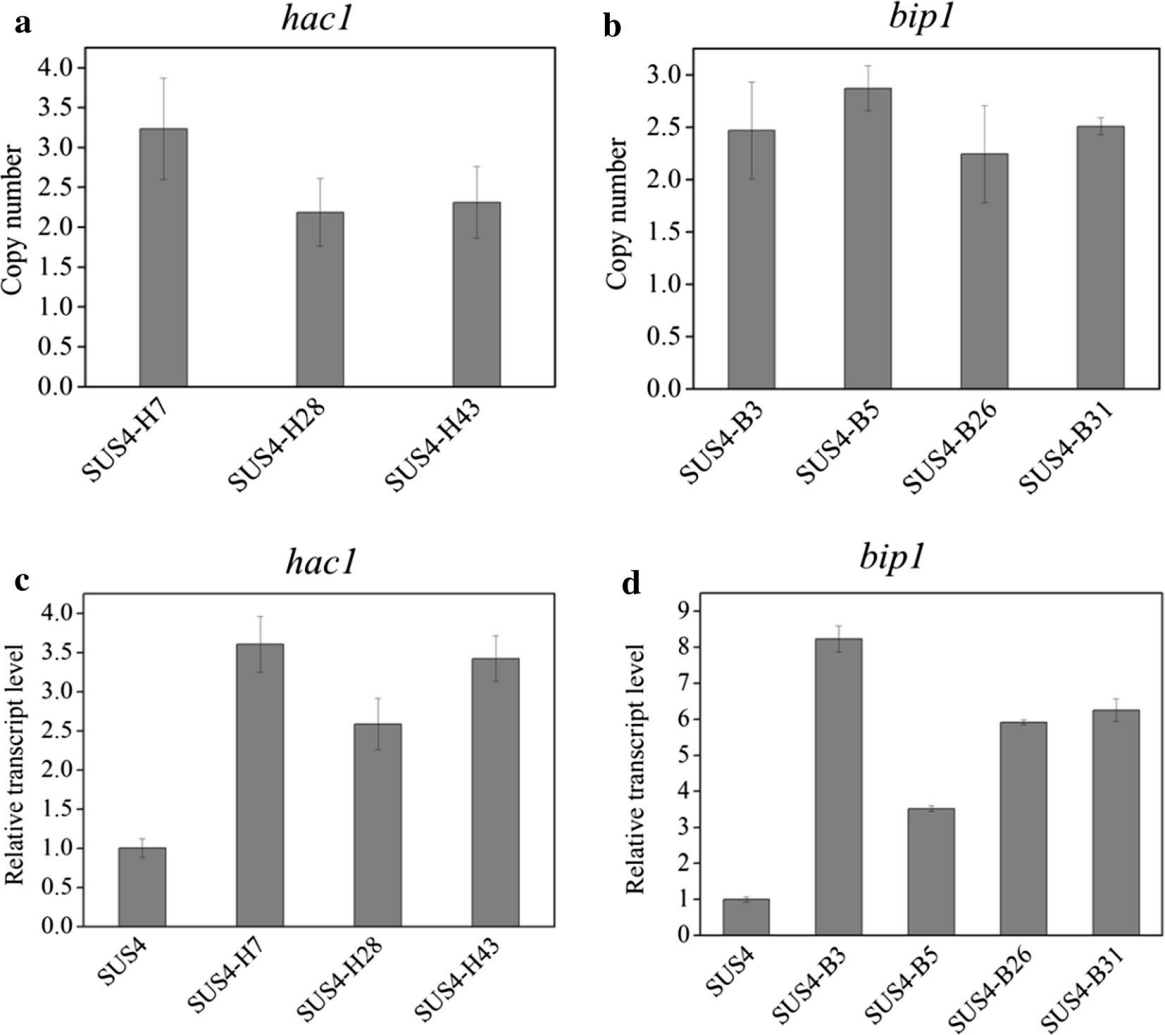
Identification of *hac1*- and *bip1*- overexpression in isolated transformants with improved cellulase-producing ability. **a**, **b** Copy numbers of *hac1* (**a**) and *bip1* (**b**) in the transformants as determined by quantitative PCR. The expressing cassettes for only *hac1* or *bip1*, but not the other four genes, were found in the seven strains with improved cellulase-producing ability. **c**, **d** The relative transcript levels of *hac1* (**c**) and *bip1* (**d**) in these transformants as analyzed by RT-qPCR

The plasmid encoding the *DsRed*-*AfMP1* chimeric gene was transformed into SUS2. The chimeric fluorescent protein was successfully expressed and correctly located because the hyphae were surrounded with red fluorescence (Fig. 4a). The display of DsRed-*Af*MP1 on *T. reesei* cell surface was further verified by sequentially incubating the germinated transformant spores with a monoclonal antibody against DsRed and FITC-labeled goat anti-mouse secondary antibody followed by flow cytometry analyses of both DsRed and FITC signals (Additional file 3). One should note that the red fluorescence of the *DsRed*-*AfMP1* transformant (SUS4) was weaker than that expressing intracellular DsRed. This was also reflected by the color change of the *DsRed*-*AfMP1*–transformant (Additional file 1). To analyze if surface-displayed DsRed could be used to screen beneficial genetic alterations at stages beyond transcription, six genes (*bip1*, *hac1*, *ftt1*, *sso2*, *sar1*, and *ypt1*) regulating cellulase folding and secretion were constructed into plasmids individually under strong constitutive promoters. Bip1 is an ER-resident molecular chaperone assisting folding of nascent proteins, while Hac1 is a global transcription regulator controlling the unfolded protein response [35]. Ftt1, Sso2, Sar1, and Ypt1 are proteins involved in diverse stages of the cellulase secretion pathway in *T. reesei* [22]. Equal amounts of the six plasmids were combined and one-time transformed into a uridine auxotroph of SUS4. The transformant spores were pooled for FACS. Sixty spores with the highest red fluorescence signal were collected (Fig. 4b). Seven representative strains were determined to produce more cellulase (Fig. 4c) and extracellular proteins (Fig. 4d). Interestingly, we discovered by PCR that these strains were transformants overexpressing *hac1* and *bip1* only but not the other four genes (data not shown). Quantitative PCR analysis indicated that 2 to 3 copies of *hac1* (Fig. 5a) and *bip1* (Fig. 5b) genes were existent in these transformants while there was only one copy in the parent strain (data not shown). The RT-qPCR analysis further proved that both the *bip1* and *hac1* genes were transcribed to levels as high as 2.5- to 3.6-fold (*hac1*, Fig. 5c) and 3.5- to 8.2-fold (*bip1*, Fig. 5d) in these transformants.

### Isolation of cellulase hyperproducers from an insertional mutagenesis library of *T. reesei* aided by surface-displayed DsRed and FACS

**Fig. 6:**
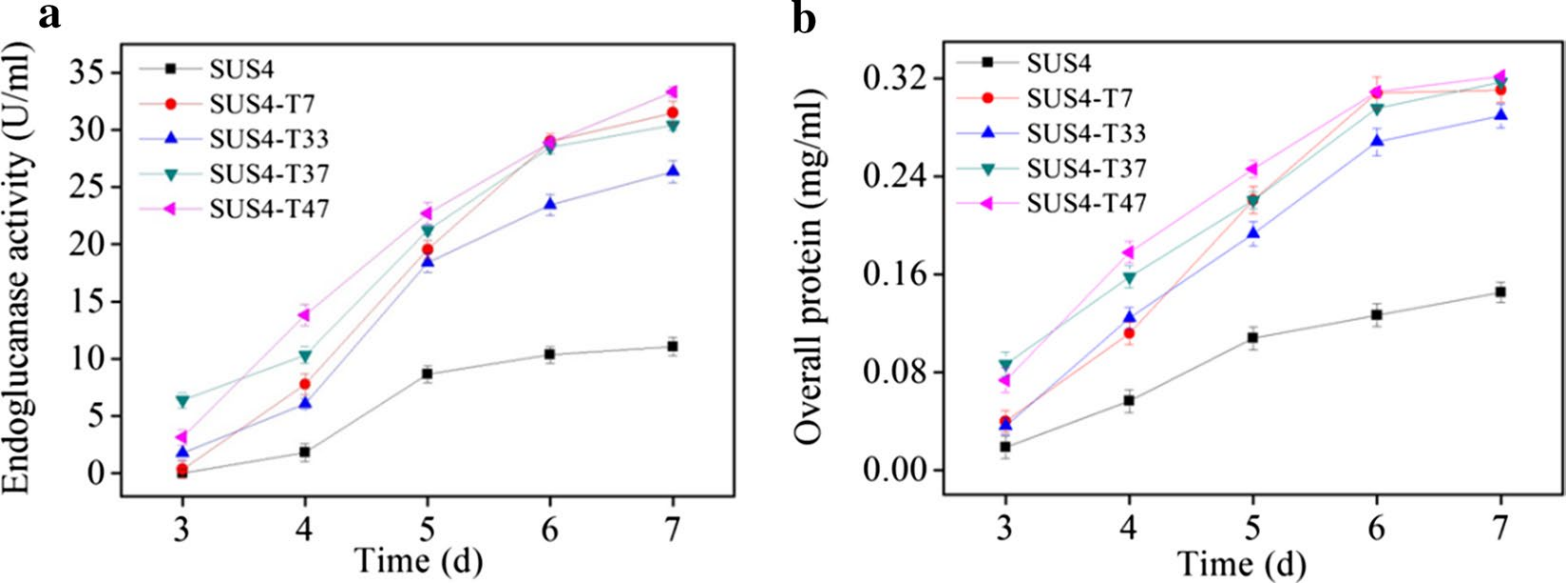
Isolation of cellulase hyperproducers from a *T. reesei* mutant library created by *Agrobacterium*-mediated random insertion of the *pyr4* gene in the chromosome. **a** Endoglucanase activity of the representative strains. **b** Extracellular protein concentrations of the representative strains

*Agrobacterium*-mediated transformation was used to randomly insert the *pyr4* gene into the chromosome of the uridine auxotroph of SUS4. It was expected that, when the function of a gene negatively affecting cellulase expression was disrupted, the recombinant strain would display a cellulase hyperproducer phenotype. The spores from the transformants were pooled, germinated in MM-lactose/sophorose, and sorted by FACS to obtain sixty spores with highest red fluorescence. In shake flask fermentation, four representative strains displayed higher endoglucanase activities (26.3–33.3 U/ml, Fig. 6a) and extracellular protein concentration (0.29–0.32 mg/ml, Fig. 6b) than those of the parent strain (11.1 U/ml and 0.15 mg/ml, respectively) at the end of fermentation.

## Discussion

As a response to the lack of easy-to-ferment sugars such as glucose, *T. reesei* secrets cellulase which enables it to acquire carbon and energy from its natural habitat. Biosynthesis of cellulase is complex: induction of cellulase in *T. reesei* is well acknowledged to entail formation of sophorose, but very likely other oligo- or mono-saccharides, as well [12]. Numerous glycoside hydrolases, metabolic enzymes, transcription factors, signaling proteins, and secretory factors have, therefore, been discovered to be involved in initiating cellulase induction. Being glycoside hydrolases, cellulase can also be regarded as a special, excreted polypeptide metabolite. This implies that the availability of amino acid precursors and the intracellular redox and metabolic balance will also have a significant impact on cellulase biosynthesis and secretion [36]. These pieces of newly uncovered knowledge present unprecedented opportunities to modify *T. reesei* for improved cellulase production. Random mutagenesis, genetic engineering (particularly multiplex genome engineering), or even directed evolution of key regulatory genes can all be potentially utilized in *T. reesei* for speeded strain improvement. However, all these endeavors require a high-throughput method as a prerequisite to isolate the cellulase hyperproducers.

The high-throughput method described herein for screening *T. reesei* cellulase hyperproducers was based on coupling the usage of DsRed and FACS. One advantage of DsRed is that the positive colonies turn to red, making them easily identified by naked eyes from the transformants (Additional file 1). Apparently, the depth of red color is positively associated with the inducing extent of *cbh1* promoter. Using this system, we demonstrated as proof-of-concept that cellulase hyperproducers could be isolated from *ace3*-transformants and from a mutagenized library. Cellulose plate assay and preliminary shake flask fermentation confirmed the effectiveness of this method with the number of colonies largely reduced to as few as ~ 50, saving tremendous time and labor than the previous screening method [14]. Despite these successes, expressing DsRed intracellularly is limited to discovery of genetic modifications majorly favoring transcription, in sharp contrast with the complex nature of cellulase expression regulation in *T. reesei* [37, 38]. Therefore, we overcome this hurdle by further developing a surface-display technology of DsRed and demonstrated that the updated method managed to isolate beneficial genetic modifications (*hac1*- and *bip1*-overexpression) favoring nascent protein folding and secretion. The overexpressing cassettes for *sso2*, *ftt1*, *sar1*, and *ypt1* could not be amplified from the isolated cellulase hyperproducers. It is possible that the expression of these genes may not be a bottleneck for cellulase expression in the investigated strain and under the specific culture condition. However, it could also be that the cellulase hyperproducers bearing one of these genes occasionally escaped the FACS isolation in the current study. These hypotheses undoubtedly need further investigations.

Different from small metabolites whose production can be either auto-regulated [39] or monitored by biosensor-based screening [40], cellulase is a secreted, special metabolite which cannot be readily quantified using any known, existent biosensors. Coupling DsRed-*Af*MP1 expression with FACS passes this barrier, enabling fast isolation of *T. reesei* cellulase hyperproducers from both engineered and mutagenized libraries. We noticed that, however, expression of DsRed-*Af*MP1 chimera reduced cellulase secretion in the host strain, suggesting that DsRed-*Af*MP1 might compete with the endogenous cellulase for the same secretory pathway. This trait is undesirable from the perspective of strain improvement. However, with this surface-display system, the impaired *T. reesei* can be quickly improved back to normal or even to a higher level of production after one or a few rounds of engineering. Moreover, once the strain is modified to reach a satisfying level of cellulase production, the expression of *DsRed* can be easily eliminated using the RNAi-mediated gene silencing [6] or gene knockout using the CRISPR/Cas9 system [41], further releasing the occupied carbon, energy, and secretion pathway components for cellulase.

At this time, the relationship of the sugar utilization metabolic pathways or redox balance to cellulase induction in *T. reesei* is still not clear, preventing us from testing the efficacy of this system to obtain cellulase hyperproducers by engineering these metabolic processes. It is also not known to what extent DsRed and cellulase share the expression and secretory machineries. The current system can thus be further improved by constructing a modified DsRed-*Af*MP1 chimeric protein by fusing DsRed to the C-terminus of a major cellulase (CBH1, CBH2, EG1, or EG2) [42], whose mode of secretion is expected to be much more similar to that of the cellulase. With slight modifications, this versatile system can also be used for strain improvement to produce heterologous proteins in *T. reesei*. Moreover, the use of the red fluorescence protein, particularly in a surface-displayed form, coupled with FACS, can also be easily implanted to other industrially important protein producing filamentous fungi such as *Aspergillus oryzae* [43], *A. niger* [44], *Neurospora crassa* [45], and *Myceliophthora thermophila* [46].

## Conclusions

In this study, we established a high-throughput method for fast isolation of *T. reesei* cellulase hyperproducers. Specifically, coupling expression of DsRed with FACS allowed us to rapidly isolate cellulase hyperproducers from *ace3*-transformants and from a random mutagenized *T. reesei* library. Furthermore, displaying DsRed on *T. reesei* cell surface enabled isolation of cellulase hyperproducers with genetic variations for enhanced expression of proteins involved in nascent protein folding and secretion (*bip1* and *hac1*) beyond transcription. This versatile system saves tremendous time and labor than the previous screening methods and can, therefore, be used as a robust engineering framework for future metabolic engineering of *T. reesei*, as well other industrially important filamentous fungi for enhanced secretion of cellulase and other valuable proteins.

## Methods

### Strains, plasmids, and culture conditions

The *Escherichia coli* Trans I-T1 strain from Transgen (Beijing, China) was used as a host for plasmid construction and propagation. The *Saccharomyces cerevisiae* AH109 (Clontech, San Francisco, CA) auxotrophic strain was used as the host for constructing the plasmids containing the expressing cassettes via DNA assembler [47]. The *T. reesei* SUS2 strain is uridine-auxotrophic and derived from SUS1 [8] by transforming extra copies of the endogenous *cel5A* (*egl2*) gene under control of the *cbh1* promoter. The *Agrobacterium tumefaciens* AGL-1 strain was used as the T-DNA donor for *A. tumefaciens*-mediated transformation (AMT) of *T. reesei*. The plasmids containing the codon-optimized *DsRed* gene (pSKLR) [25] and the *AfMP1* gene (pGFP-Mp1) [32] are kindly gifts from Prof. Zhiyang Dong and Haomiao Ouyang, respectively, from the Institute of Microbiology Chinese Academy of Sciences. The plasmid pTi was provided by Dr. XinXin Xu from Biotechnology Research Institute, Chinese Academy of Agricultural Sciences.

The *E. coli* and *A. tumefaciens* were cultured in the Luria–Bertani (LB) medium supplemented with an appropriate antibiotic when needed. The yeast AH109 strain was cultivated in yeast peptone dextrose medium with adenine (YPDA) at 30 °C. *T. reesei* was grown on potato dextrose agar (PDA) at 28 °C for sporulation. For cellulase production, *T. reesei* was first grown in minimal medium (MM) ((NH_4_)_2_SO_4_, 5.0 g/L; KH_2_PO_4_, 15 g/L; MgSO_4_, 0.6 g/L; CaCl_2_, 0.6 g/L; FeSO_4_·7H_2_O, 0.005 g/L; MnSO_4_·H_2_O, 0.0016 g/L; ZnSO_4_·7H_2_O, 0.0014 g/L; CoCl_2_, 0.002 g/L) supplemented with 2% glucose as the sole carbon source and then mycelia were transferred to the MM-2% Avicel medium. The basal medium (BM), induction medium (IM), and co-cultivation medium (CM) used for AMT were prepared as described previously [48].

### Plasmid construction

The DNA assembler method which utilizes the highly efficient *in* *vivo* homologous recombination machinery was employed in this study to construct the *DsRed*-expressing plasmid. The *DsRed* gene was amplified from pSKLR using the primer pairs DsRedF/R (Additional file 4). The *cbh1* promoter and terminator were amplified from the genomic DNA of TU-6 using the Trcbh1pF/R and Trcbh1tF/R primer pairs, respectively (Additional file 4). The *Eco*RI-linearized pRS424 plasmid was mixed with the *cbh1* promoter, *DsRed*, and *cbh1* terminator and co-transformed into *S. cerevisiae* AH109 to obtain pRS-DsRed. The *DsRed* expressing cassette was amplified from pRS-DsRed using the primers DsRedcbh1F/R and inserted into the *Eco*RI and *Bam*HI sites of the plasmid pAPA by Gibson assembly [49], which was constructed by inserting a marker gene *pyr4* flanked by two tandemly repeated ampicillin resistance genes [8]. This resulted into pCbh1-DsRed for intracellular expression of DsRed. The direct repeats of ampicillin resistance genes were used for looping out the *pyr4* selection marker gene in the *T. reesei* transformants when needed [8, 50]. For surface-display of DsRed, the *AfMP1* GPI anchor gene was first fused to the 3′-end of *DsRed* gene (containing the gene encoding the *cbh1* signal peptide at the 5′-end) by overlap extension PCR using the primers SPcbh1-DsRedF/R and MP1F/R, respectively (Additional file 4). Then the *DsRed*-*AfMP1* gene was assembled with the *cbh1* promoter and terminator and inserted into the pAPA plasmid to obtain pDsRed-AfMP1. The pPdc1-ace3 plasmid was constructed by the same method using the *pdc1* promoter and terminator (Table 1, Additional file 2). The expressing cassettes of six genes (*sso2*, *ftt1*, *sar1*, *ypt1*, *bip1*, and *hac1*) (Table 1) involved in nascent protein folding and secretion were amplified from our previously constructed plasmids [7] and ligated into pAPA. For AMT-mediated insertional mutagenesis of *T. reesei*, the *pyr4* gene was amplified from the genomic DNA of QM9414 with the primer pairs pyr4F/R (Additional file 4) and ligated into the *Xma*JI and *Pac*I restriction sites of the plasmid pTi using the ClonExpress^®^ II One Step Cloning Kit (Vazyme, Nanjing, China) to obtain the plasmid pTi-pyr4 (Additional file 5).

**Table 1:** The regulatory genes involved in cellulase expression in *T. reesei* under investigation in this study

Gene	Function	Promoter^a^	Terminator^a^	References
*ace3*	Transcription activator	*pdc1*	*pdc1*	[22]
*bip1*	Chaperone	*eno1*	*eno1*	[27]
*hac1*	Unfolded protein response	*eno1*	*eno1*	[35]
*ftt1*	Secretion	*pdc1*	*pdc1*	[54]
*sso2*	t-SNARE protein	*pdc1*	*pdc1*	[22]
*sar1*	Vesicle budding	*gpd1*	*gpd1*	[55]
*ypt1*	Vesicle fusion	*gpd1*	*gpd1*	[56]

^a^The promoters and terminators used to control gene expression in this study

### Transformation of *T. reesei*

The plasmids were introduced into *T. reesei* using the polyethylene glycol (PEG)-mediated protoplast transformation method [51]. Briefly, *T. reesei* was grown in MM-glucose (2%) at 30 °C for 24 h. The young mycelia were collected, mixed with 10 mg/ml of Lysing Enzymes from *Trichoderma harzianum* (L1412, Sigma-Aldrich, St. Louis, MO), and frequently observed under microscope until large amounts of protoplasts were released. The uridine autotroph transformants were selected on MM-glucose plates and screened for integration of the expressing cassette into the chromosome of *T. reesei* by PCR.

*Agrobacterium*-mediated transformation was used for insertional mutagenesis of *T. reesei* [52]. Briefly, *A. tumefaciens* containing the binary vector pTi-pyr4 was grown at 28 °C for 2 days in liquid BM supplemented with kanamycin (50 μg/ml). The bacterial cell suspensions were diluted to an optical density at 600 nm (OD_600_) of 0.2 in IM with 200 μM acetosyringone (AS) and grown for 6 h to an OD_600_ of 0.4–0.8. Equal volumes of *A. tumefaciens* cells and the *T. reesei* protoplasts (10^7^/ml) were mixed. Next, 200 μl of this blend were plated on a 90-mm diameter cellophane paper on top of CM-agar in presence of 200 μM AS. After co-cultivation at 25 °C for 48 h, the cellophane paper was transferred to the selection medium (BM-agar supplemented with 1 M sorbitol and 200 μg/ml cefotaxime but lacking uridine). The transformant colonies were transferred onto PDA for sporulation. The spores were mixed and used for FACS screening.

### Verification of DsRed-*Af*MP1 expression on *T. reesei* cell surface

The *T. reesei* SUS2 and its *DsRed*-*AfMP1* transformant spores were cultured in MM-lactose/sophorose (2% for lactose and 0.003% for sophorose, w/v) at 28 °C with agitation at 200 rpm for 12 h. The germinated spores were harvested by centrifugation at 10,000 rpm and washed twice with phosphate-buffered saline (PBS) containing 1 mg/ml bovine serum albumin (BSA). Cells were re-suspended in PBS containing 5 μg/ml of mouse anti-DsRed monoclonal antibody (Abcam, Shanghai, China) and incubated at room temperature for 2 h. The cells were washed again with PBS and incubated with 10 μg/ml of fluorescein isothiocyanate (FITC)-labeled goat anti-mouse IgG secondary antibody (Abcam) for 1 h. The cells were finally washed twice with PBS and passed through flow cytometry for analyses of both DsRed and FITC signals.

### ARTP-mediated mutagenesis of *T. reesei*

For ARTP-mediated mutagenesis, *T. reesei* spores harvested from 5-day-old culture of the strain SUS3-A27 on PDA plate were suspended in distilled water to a concentration of 10^9^ per ml. Ten μl of this spore suspension were spread on the surface of a sterilized sample plate and subjected to ARTP treatment. The radio-frequency power input was set as 100 W and the helium gas flow rate was 10 SLM (standard liter per minute) with a plasma action distance of 2 mm. The processing time was set at 3 min. After ARTP treatment, the spores were transferred to 1 ml of distilled water. The spores were plated on PDA plates and allowed to grow for re-sporulation for 5–7 days. Finally, spores from these plates were mixed for FACS.

### FACS

Fresh spores were inoculated into liquid MM-lactose/sophorose (2% for lactose and 0.003% for sophorose, w/v) and cultured at 28 °C with vigorous shaking for 14 h. High-speed sorting was performed on a FACS Aria sorter at a rate of 5000 events per second, 30 psi with an 85 μm nozzle. Single germlings with the brightest (top 0.1%) DsRed signal were sorted into individual wells of 6-well plates and incubated at 28 °C for sporulation.

### Induction of cellulase expression in *T. reesei*

For shake flask fermentation, fresh spores (1 × 10^7^) of *T. reesei* were individually inoculated into 50 ml of liquid MM-glucose (2%) and cultured at 28 °C with agitation at 200 rpm for 48 h. The mycelia were collected and washed twice with MM to remove residual glucose. One gram of the mycelia was then transferred to 100 ml of MM-Avicel (2%) for cellulase induction. The culture was continued at 28 °C for 7 days. From 72 h to 168 h post-induction, the culture supernatants were periodically sampled for assay of the cellulase activities and protein concentrations.

### Assay of endoglucanase activity and protein concentration

For assay of the endoglucanase activity, 900 μl of 1% (w/v) sodium carboxymethyl cellulose (CMC-Na, from Sigma-Aldrich) in the McIlvaine buffer (pH 5.0) were mixed with 100 μl of appropriately diluted enzymes. The reaction was incubated at 50 °C for 10 min and the released reducing sugars were determined using the 3,5-dinitrosalicylic acid (DNS) method. One unit of endoglucanase activity was defined as the amount of enzyme that liberated 1 μmol of reducing sugar per minute. The protein concentration was determined using the BCA-200 Protein Assay Kit (Pierce, Rockford, IL).

### Fungal growth and microcrystalline cellulose hydrolysis

The *T. reesei* spores (3 × 10^5^) were individually spotted on agar plates containing MM-Avicel (2%) and incubated at 28 °C for 4–7 days until halo around the colony could be clearly visualized. The halo diameters were measured and compared.

### Reverse transcription quantitative PCR analysis

For reverse transcription quantitative PCR (RT-qPCR), the mycelia of *T. reesei* cultured in MM-Avicel (2%) for 24 h were collected and pulverized in liquid nitrogen using a pestle and mortar. The total RNA was extracted using the TRIzol reagent (Thermo Fisher Scientific, Waltham, MA). The cDNA was synthesized using the First Strand cDNA Maxima Synthesis kit (TOYOBO, Shanghai, China). RT-qPCR was performed in an Applied Biosystems™ QuantStudi™ 6 Flex Real-Time PCR System (Applied Biosystems, San Diego, CA) using a TransScript Green One-Step SuperMix (TransGen, Beijing, China). The *actin* gene was used as an endogenous reference. The primers used for RT-qPCR were listed in Additional file 4. The following amplification conditions were used: 95 °C for 10 min for initial denaturation, 40 cycles of 94 °C for 10 s, 60 °C for 20 s, and 72 °C for 20 s.

### Determining copy numbers by qPCR

To determine the copy numbers of the integrated *ace3*, *bip1* or *hac1* gene in the transformants, the genomic DNA was extracted from the mycelia by a Fungal DNA kit (Omega bio-tek, USA) and used as the template for quantitative PCR (qPCR). The qPCR method was performed as that described by Solomon [53]. The *cbh1* gene was used to represent a single copy gene. The qPCR was performed with the SYBR Green Real-time PCR Master Mix (TOYOBO, Osaka, Japan) in a CFX96 Real-Time PCR Detection System (Bio-Rad, Hercules, CA). The primers used for qPCR were also listed in Additional file 4. The qPCR conditions were: 94 °C for 30 s, 40 cycles of 94 °C for 30 s, 62 °C for 30 s, and 72 °C for 20 s.

## Additional files

**Additional file 1.** Observation of representative *T. reesei* strains expressing *DsRed* by naked eyes. The culture medium is MM-lactose plus agar.

**Additional file 2.** Schematic diagram of pPdc1-ace3.

**Additional file 3.** Verification of DsRed-*Af*MP1 expression on *T. reesei* cell surface.

**Additional file 4.** Primers used in this study.

**Additional file 5.** Schematic diagram of pTi-pyr4. *LB* left border, *RB* right border.

## Abbreviations

FACS: fluorescence-assisted cell sorting
GFP: green fluorescence protein
AMT: *Agrobacterium tumefaciens*-mediated transformation
YPDA: yeast extract-peptone dextrose medium with adenine
PDA: potato dextrose agar
MM: minimal medium
BM: basal medium
IM: induction medium
CM: co-cultivation medium
AS: acetosyringone
ARTP: atmospheric and room temperature plasma
DNS: 3,5-dinitrosalicylic acid
CBH1: cellobiohydrolase I
CBH2: cellobiohydrolase II
EG1: endoglucanase I
EG2: endoglucanase II
GPI: glycosylphosphatidylinositol
SLM: standard liter per minute
