# Supplementary material for: A versatile system for fast screening and isolation of Trichoderma reesei cellulase hyperproducers based on DsRed and fluorescence-assisted cell sorting
Source: Biotechnol Biofuels. 2018 Sep 24;11:261. doi: 10.1186/s13068-018-1264-z (PMC6151939; doi:10.1186/s13068-018-1264-z)
Supplement: Supplementary file 1 — Additional file 1. Observation of representative T. reesei strains expressing DsRed by naked eyes. The culture medium is MM-lactose plus agar. [file 13068_2018_1264_MOESM1_ESM.docx]

**
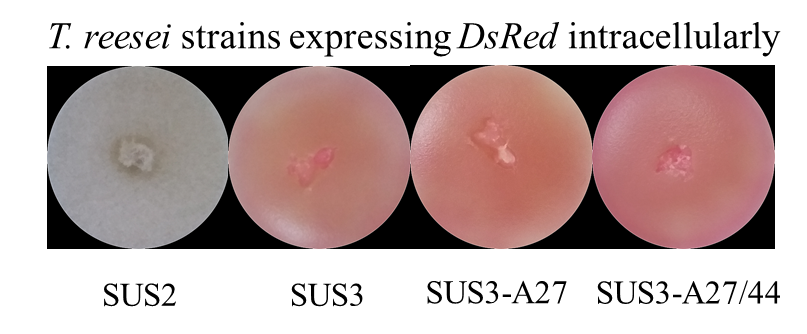
**

**
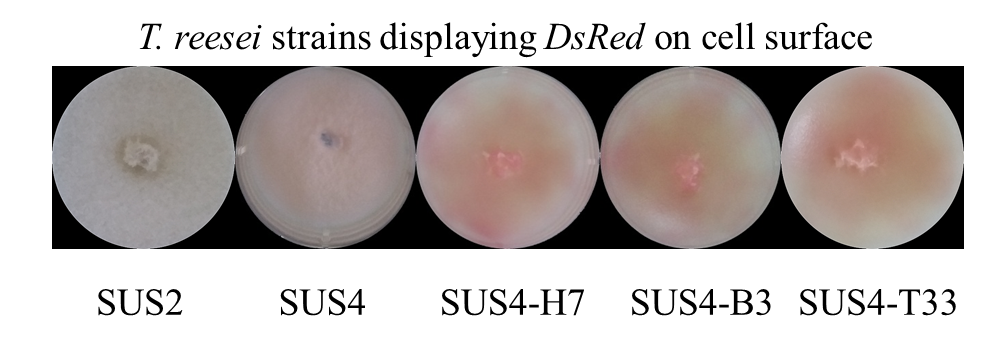
**

**Additional file 1.** Observation of representative *T. reesei* strains expressing *DsRed* by naked eyes. The culture medium is MM-lactose plus agar.
