# Supplementary figures and images for: A versatile system for fast screening and isolation of Trichoderma reesei cellulase hyperproducers based on DsRed and fluorescence-assisted cell sorting
Source: Biotechnol Biofuels. 2018 Sep 24;11:261. doi: 10.1186/s13068-018-1264-z (PMC6151939; doi:10.1186/s13068-018-1264-z)

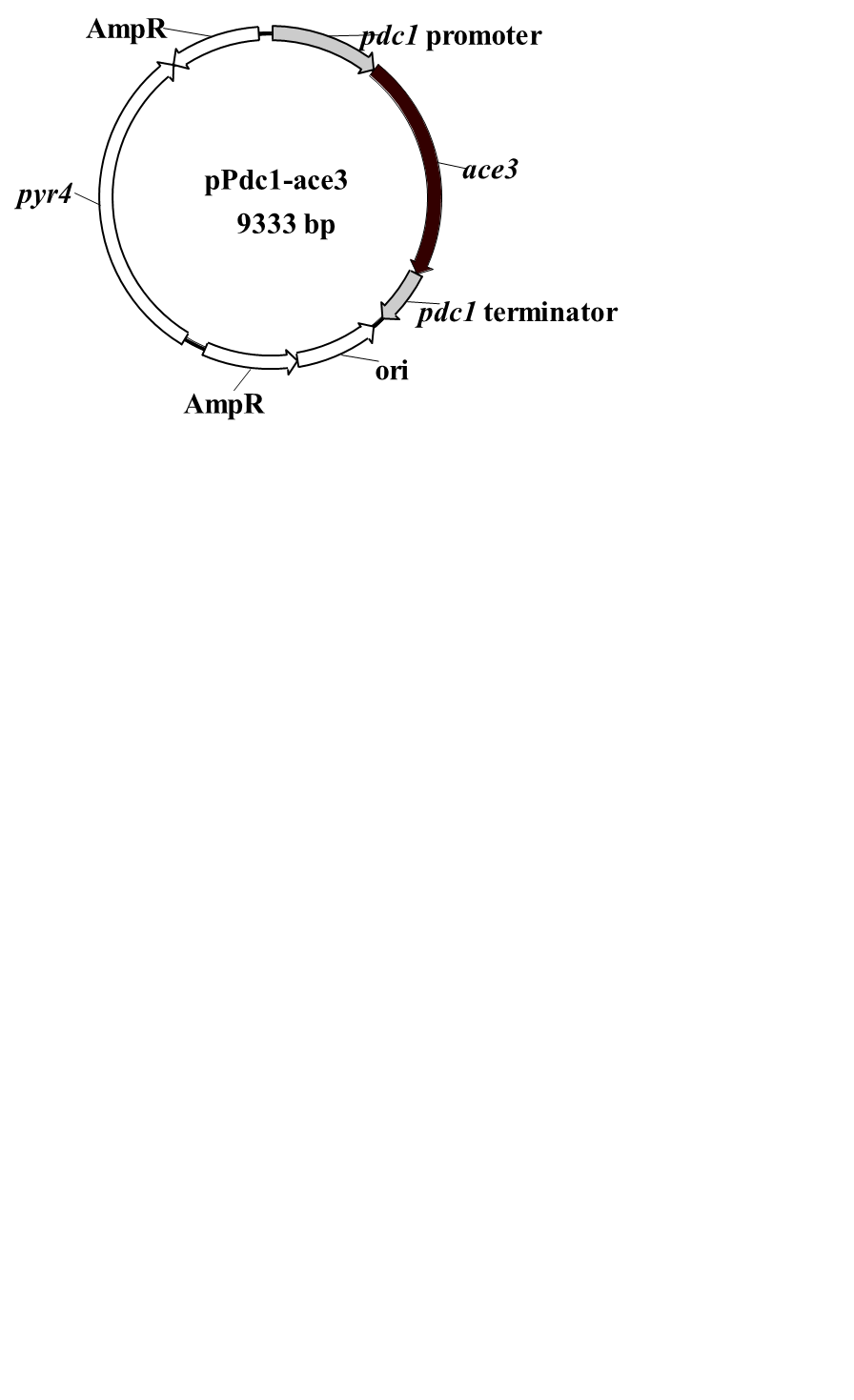


**Additional file 2.** Schematic diagram of pPdc1-ace3.

Supplement: Supplementary file 2 — Additional file 2. Schematic diagram of pPdc1-ace3. [file 13068_2018_1264_MOESM2_ESM.docx]

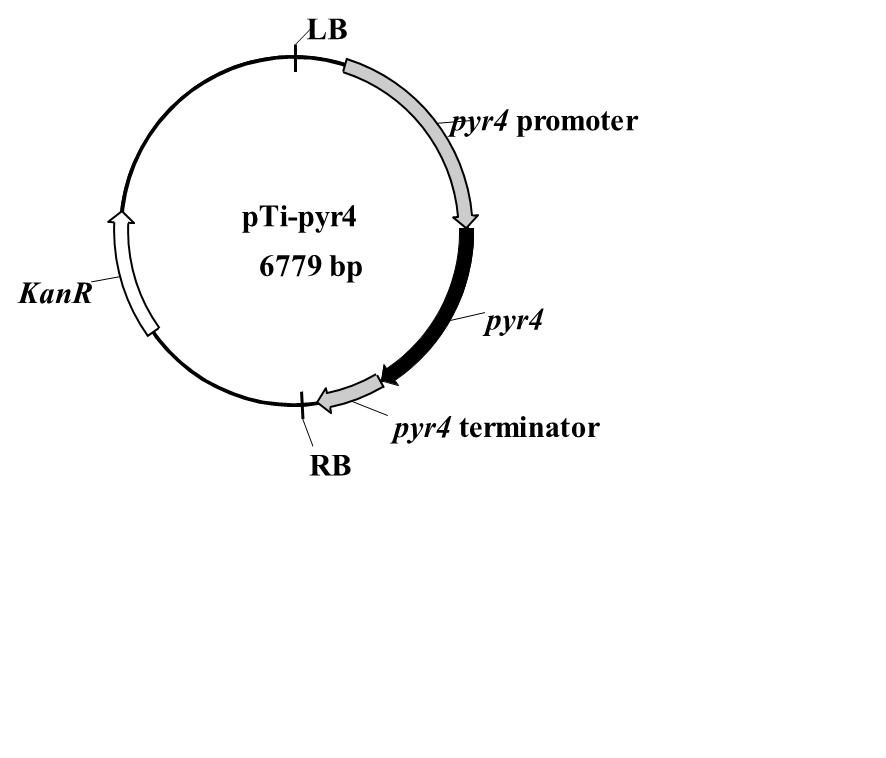


**Additional file 5.** Schematic diagram of pTi-pyr4. LB: left border; RB: right border.

Supplement: Supplementary file 5 — Additional file 5. Schematic diagram of pTi-pyr4. LB left border, RB right border. [file 13068_2018_1264_MOESM5_ESM.docx]
