# Supplementary material for: A versatile system for fast screening and isolation of Trichoderma reesei cellulase hyperproducers based on DsRed and fluorescence-assisted cell sorting
Source: Biotechnol Biofuels. 2018 Sep 24;11:261. doi: 10.1186/s13068-018-1264-z (PMC6151939; doi:10.1186/s13068-018-1264-z)
Supplement: Supplementary file 3 — Additional file 3. Verification of DsRed-AfMP1 expression on T. reesei cell surface. [file 13068_2018_1264_MOESM3_ESM.docx]

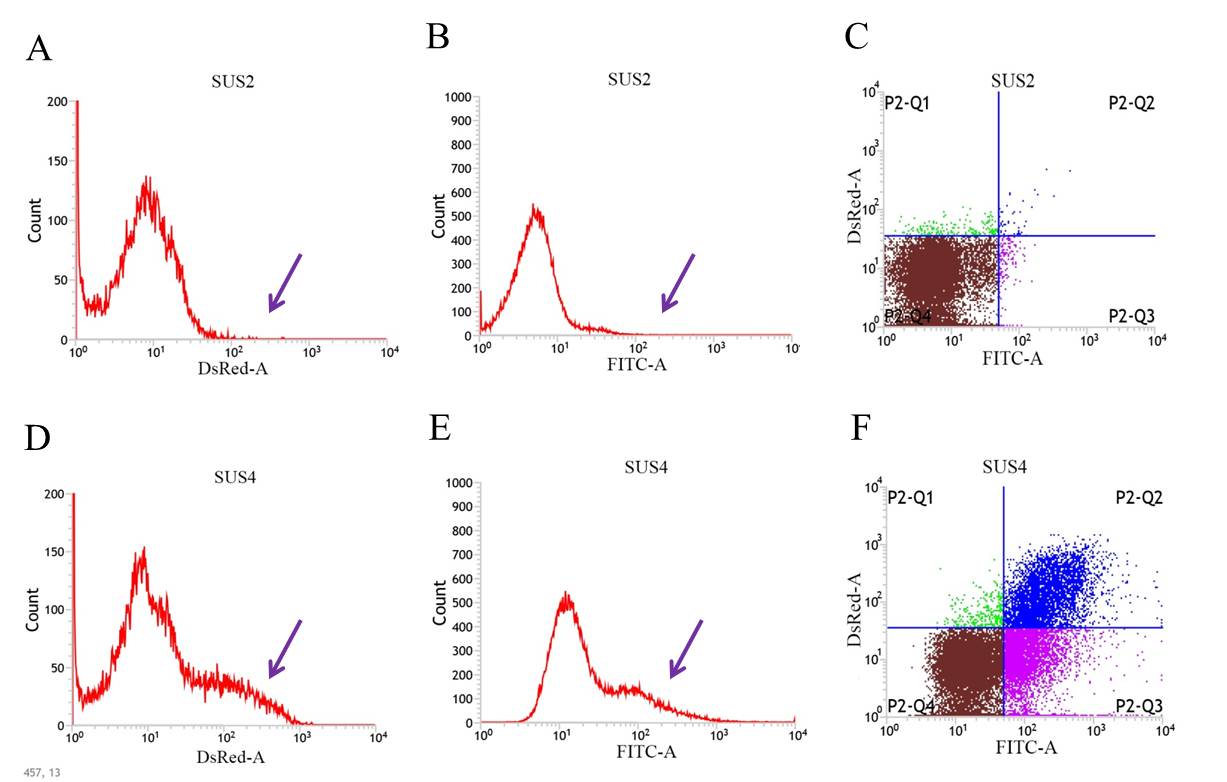


**Additional file 3.** Verification of DsRed-*Af*MP1 expression on *T. reesei* cell surface. The SUS2 and SUS4 spores were germinated in MM-lactose/sophorose for 12 h and sequentially reacted with mouse anti-DsRed monoclonal antibody and FITC-labeled goat anti-mouse IgG secondary antibody. The cells were washed with phosphate buffered saline and checked for DsRed (A, D), FITC (B, E), or both (C, F) signals by flow cytometry analysis. The arrows indicate the difference in DsRed and FITC signals in the two strains.
