# Supplementary material for: A versatile system for fast screening and isolation of Trichoderma reesei cellulase hyperproducers based on DsRed and fluorescence-assisted cell sorting
Source: Biotechnol Biofuels. 2018 Sep 24;11:261. doi: 10.1186/s13068-018-1264-z (PMC6151939; doi:10.1186/s13068-018-1264-z)
Supplement: Supplementary file 4 — Additional file 4. Primers used in this study. [file 13068_2018_1264_MOESM4_ESM.docx]

**Additional file 4. Primers used in this study**

| Primer | Sequence 5'-3' | Usage |
| --- | --- | --- |
| DsRedF | ATGGACAACACCGAGGACGT | Amplification of *DsRed* for construction of pRS-DsRed |
| DsRedR | CTACTGGGAGCCGGAGTGGC | Amplification of *DsRed* for construction of pRS-DsRed |
| Trcbh1pF | gcgcgtaatacgactcactatagggcgaattggcggccgcgggtttggagcaatgtggga | Amplification of *cbh1* promoter for construction of pRS-DsRed and pRS-DsRed-AfMP1 |
| Trcbh1pR | ACTGCATGAACTCCTTGATGACGTCCTCGGTGTTGTCCATgatgcgcagtccgcggttga | Amplification of *cbh1* promoter for construction of pRS-DsRed |
| Trcbh1tF | GTACGAGCACGCCGAGGCCCGCCACTCCGGCTCCCAGTAGagctccgtggcgaaagcctg | Amplification of *cbh1* terminator for construction of pRS-DsRed |
| Trcbh1tR | caattaaccctcactaaagggaacaaaagctggcggccgcaacacttcggtggaggtgtc | Amplification of *cbh1* terminator for construction of pRS-DsRed and pRS-DsRed-AfMP1 |
| DsRedcbh1F | GGGTTAATTGCGCGCTTGGCtcaacctttggcgtttccctgattc | Amplification of *cbh1* promoter-*DsRed*-*cbh1* terminator for construction of pCbh1-DsRed; amplification of *cbh1* promoter-*DsRed-AfMP1-cbh1* terminator for construction of pDsRed-AfMP1 |
| DsRedcbh1R | TGATTCTGTGGATAACCGTAaatttccactgttgctattatgctg | Amplification of *cbh1* promoter-*DsRed*-*cbh1* terminator for construction of pCbh1-DsRed; amplification of *cbh1* promoter-*DsRed-AfMP1-cbh1* terminator for construction of pDsRed-AfMP1 |
| SPcbh1-DsRedF | atgtatcggaagttggccgtcatctcggccttcttggccacagctcgtgctATGGACAACACCGAGGACGTCATC | Amplification of *cbh1 signal peptide-DsRed* gene for construction of pDsRed-AfMP1 |
| SPcbh1-DsRedR | CggagccgccCTGGGAGCCGGAGTGGCGGGCCTCG | Amplification of *cbh1 signal peptide-DsRed* gene for construction of pDsRed-AfMP1 |
| MP1F | CGGCTCCCAGggcggctccggctccggctccggc | Amplification of *AfMP1* for construction of pDsRed-AfMP1 |
| MP1R | ttagagagcgacggcgatggcggccgc | Amplification of *AfMP1* for construction of pDsRed-AfMP1 |
| Trcbh1p1R | tggccaagaaggccgagatgacggccaacttccgatacatgatgcgcagtccgcggttga | Amplification of *cbh1* promoter for construction of pRS-DsRed-AfMP1 |
| Trcbh1t1F | cggtgccgtcgtcgcggccgccatcgccgtcgctctctaaagctccgtggcgaaagcctg | Amplification of *cbh1* terminator for construction of pRS-DsRed-AfMP1 |
| Ace3F | ATGCTGCGCTACTCCCCCGTCTTAC | Amplification of *ace3* for construction of pRS-ace3 |
| Ace3R | TTAGCCAACAACGGTAGTGGACGTA | Amplification of *ace3* for construction of pRS-ace3 |
| Trpdc1pF | gcgcgtaatacgactcactatagggcgaattggcggccgcCGATGAAAGCCTTGCAACTGTGGTG | Amplification of *pdc1* promoter for construction of pRS-ace3 |
| Trpdc1pR | AGAGAGTATCCAGGTGTAAGACGGGGGAGTAGCGCAGCATGATTGTGCTGTAGCTGCGCTGCTTT | Amplification of *pdc1* promoter for construction of pRS-ace3 |
| Trpdc1tF | TACGTCCACTACCGTTGTTGGCTAACCCGGCATGAAGTCTGACCGGGTAGTATGAGGGTTCATCG | Amplification of *pdc1* terminator for construction of pRS-ace3 |
| Trpdc1tR | caattaaccctcactaaagggaacaaaagctggcggccgcTCTTAGGAGCGGAATCTGACATTGC | Amplification of *pdc1* terminator for construction of pRS-ace3 |
| Ace3pdc1F | GGGTTAATTGCGCGCTTGGCCGATGAAAGCCTTGCAACTGTGGTG | Amplification of *pdc1* promoter-*ace3*-*pdc1* terminator for construction of pPdc1-ace3 |
| Ace3pdc1R | TAACCGTAGAATTCaatttcTCTTAGGAGCGGAATCTGACATTGC | Amplification of *pdc1* promoter-*ace3*-*pdc1* terminator for construction of pPdc1-ace3 |
| Ftt1Sso2F | AGGGTTAATTGCGCGCTTGGCGGTGTCGAGCCGGGAGGAGTTTTCGC | Amplification of *pdc1* promoter-*Sso2-pdc1* terminator for construction of pAPA-sso2; amplification of *pdc1* promoter-*ftt1-pdc1* terminator for construction of pAPA-ftt1 |
| Ftt1Sso2R | CCTGATTCTGTGGATAACCGTACGTCGGCCGGGTGGTGAGCTTCTGA | Amplification of *pdc1* promoter-*Sso2-pdc1* terminator for construction of pAPA-sso2; amplification of *pdc1* promoter-*ftt1-pdc1* terminator for construction of pAPA-ftt1 |
| Sar1Ypt1F | AGGGTTAATTGCGCGCTTGGCGCCCGGTGGAGAGAGGGAAAAAGAAG | Amplification of *gpd1* promoter-*sar1-gpd1* terminator for construction of pAPA-sar1; amplification of *gpd1* promoter-*ypt1-gpd1* terminator for construction of pAPA-ypt1 |
| Sar1Ypt1R | CCTGATTCTGTGGATAACCGTAACAATGTAATTTTCAACGAGACTCT | Amplification of *gpd1* promoter-*sar1-gpd1* terminator for construction of pAPA-sar1; amplification of *gpd1* promoter-*ypt1-gpd1* terminator for construction of pAPA-ypt1 |
| Bip1Hac1F | AGGGTTAATTGCGCGCTTGGCGTTCTCAAATACCGCAGAGGCGACAT | Amplification of *eno1* promoter-*bip1-eno1* terminator for construction of pAPA-bip1; amplification of *eno1* promoter-*hac1-eno1* terminator for construction of pAPA-hac1 |
| Bip1Hac1R | CCTGATTCTGTGGATAACCGTATTCTGTGTACTCTGTACTTTGACCG | Amplification of *eno1* promoter-*bip1-eno1* terminator for construction of pAPA-bip1; Amplification of *eno1* promoter-*hac1-eno1* terminator for construction of pAPA-hac1 |
| pyr4F | CCCGGGCTGGCCACGGCCGCGACTGACCCCCCCGGTTGGGCCCCT | Amplification of *pyr4* for construction of pTi-pyr4 |
| pyr4R | TGCATGCCTGCAGGTCGACATTACGATATCAAGCTTATCGATCAACTGCAT | Amplification of *pyr4* for construction of pTi-pyr4 |
| RTQactF | TGAGAGCGGTGGTATCCACG | RT-qPCR for *actin* |
| RTQactR | GGTACCACCAGACATGACAATGTTG | RT-qPCR for *actin* |
| RTQhacF | ACAACGTCCTGCAGTGTCAA | RT-qPCR and qPCR for *hac1* |
| RTQhacR | TAGCGATCTGCATCAAGGGC | RT-qPCR and qPCR for *hac1* |
| RTQbipF | Aagaaggttacccacgccg | RT-qPCR and qPCR for *bip1* |
| RTQbipR | Atcaaaggtaccaccaccgag | RT-qPCR and qPCR for *bip1* |
| Qace3F1 | TTTCTGCACACCCTTTTCTTCG | qPCR for *ace3* |
| Qace3R1 | AAGGGTGAATCCTGGTTGCG | qPCR for *ace3* |
| Qcbh1F6 | ATTCGGCGGATCCTCTTTCTC | qPCR for *cbh1* |
| Qcbh1R6 | TGTGGAGGAGGTCTCGTTTGTC | qPCR for *cbh1* |
